# Case study of Appendiceal Carcinoid during Pregnancy

**Published:** 2012-09-25

**Authors:** C Poiana, M Carsote, R Trifanescu, D Terzea, A Croitoru

**Affiliations:** * ”Carol Davila” University of Medicine and Pharmacy, Bucharest, Romania; ** ”CI Parhon” National Institute of Endocrinology, Bucharest, Romania; *** ”Babes” Institute of Research and Development, Bucharest, Romania; **** ”Fundeni” Institute of Digestive Diseases and Liver Transplantation, Bucharest, Romania

**Keywords:** appendix, carcinoid, pregnancy

## Abstract

The carcinoid tumor of the appendix is one of the most common tumors of the appendix, but one of the rarest anatomic locations of carcinoids. The prognosis is very good, as most tumors are incidentally discovered during surgery for acute or sub-acute appendicitis. The diagnosis is exceptional when combined with pregnancy. We present such a case of a 27-year-old female patient. An emergency appendectomy was performed, and soon after, pregnancy was confirmed. The patient had a tumor smaller than 1 cm in diameter, at the tip of the appendix. The immunochemistry revealed the neuroendocrine profile by positive reaction for chromogranin A and synaptophysin, with a ki-67 profile at an undetectable level. After surgery, the neuroendocrine markers and the octreoscan were negative, consequently indicating a favorable prognosis. Further follow-up is necessary, even though not all the authors recommend it (considering the low index to the associated metastases, especially for small appendiceal tumors). A short review of the literature is presented, starting with this case report.

## Introduction

Appendiceal tumors vary from very aggressive adenocarcinomas to benign tumors [**1**]. Neuroendocrine tumors are closer to the last category. The appendix carcinoid tumor has a low aggressive profile. Rarely, there is an associated carcinoid syndrome. The most important step in the diagnosis of the appendix carcinoid is the post-surgical pathological exam, which incidentally reveals the tumor. After surgery, a follow-up of the neuroendocrine markers might be useful. 

The diagnosis of the appendiceal carcinoid tumor during pregnancy is a rare situation. The acute appendicitis picture caused by the tumor needs to be differentiated in a complicated or uncomplicated pregnancy. These cases are extremely rare.

## Case report

**Fig. 1:**
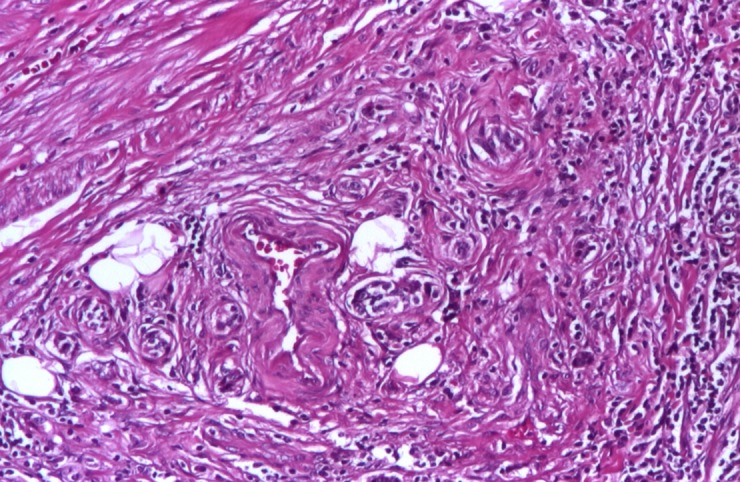
Appendix carcinoid-hematoxylin-eosin aspect 100×

A 27-year-old female patient, who was complaining of right lower quadrant abdominal pains, acute appendicitis-like, was admitted in December 2008. She had no significant medical history. She was diagnosed with acute appendicitis and immediately underwent surgery. The pathological exam revealed a tumor smaller than 1 cm in diameter, at the tip of the appendix, with no base or middle portion invasion (suggestive for a carcinoid tumor - **Fig. 1**).

**Fig. 2:**
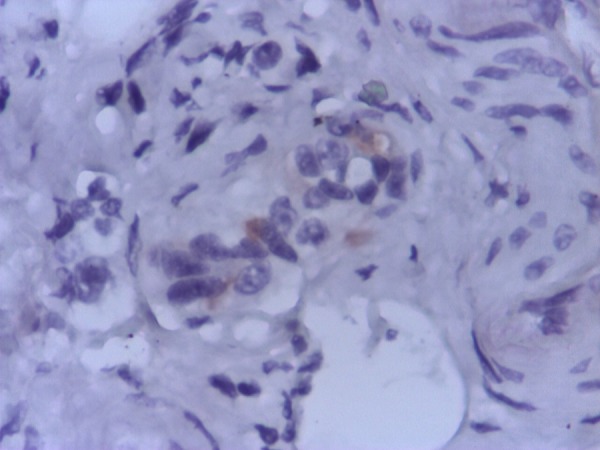
Immunohistochemistry-positive tumoral reaction for chromogranin A 400×

**Fig. 3:**
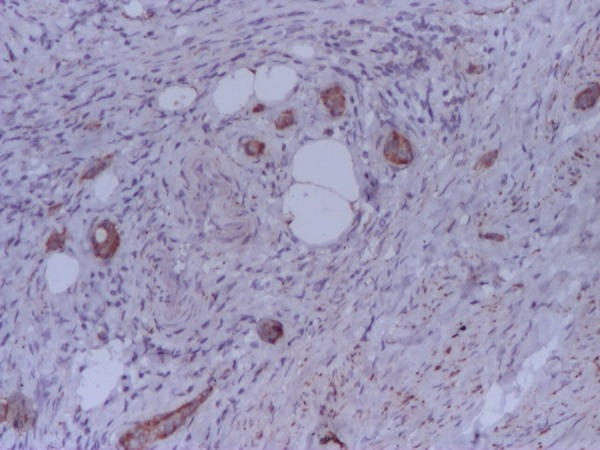
Immunohistochemistry-positive tumoral reaction for synaptophysin A 100×

The immunohistochemistry unveiled a positive reaction for chromogranin A, synaptophysin (**Figure 2,3**). The Ki-67 protein was undetectable. 

One month after surgery, pregnancy was confirmed. Due to the low aggressiveness of the tumor, as revealed by the pathological report, the patient had a good prognosis, but she decided to have an abortion. Three months after surgery, the serum neuroendocrin markers were normal: chromogranin A 40 pg/mL (normal values: 40 -100 pg/mL), and serotonin 186ng/mL (normal values: 40-200 ng/mL). The urinary 5-hydroxy indol-acetic acid was normal as well: 5.2mg/24h (normal values: 2-9). The neuron-specific enolase was also in normal ranges: 3.5µg/dl (normal value< 18.3). Although there was no specific indication at that moment, the 111 Indium Octreotide scan was performed at the patient’s request. As expected, the scan was negative. Six months later, the serum markers were still normal (chromogranin A - 36 ng/mL, serotonin -183ng/mL). Up to that point, neither the colonoscopy nor the computed tomography scans were necessary. However, we considered useful a further measurement of the neuroendocrine markers.

## Discussion

In the present case, the tumor was an incidental finding during an appendectomy. In most cases (80%), all the appendiceal carcinoids are incidentally discovered in the removed organ; there are no particular signs before surgery. Sometimes the symptoms mimic a pregnancy. The diagnosis of acute appendicitis is more difficult in pregnant women [**2**]. 

The first malignant tumor of the appendix was described in 1882, and the first reports about appendiceal tumors and pregnancy date from 1965 [**3,4**]. The incidence of cancer in pregnancy is of 1 in 1000. The most common cancers associated with pregnancy are cervical, breast and ovarian, while appendiceal tumors are extremely rare [**5**]. Most of the appendiceal carcinoids are diagnosed in adults. The median age is of 29.8 years [**6**]. Appendiceal carcinoids account for 1.8% of all intestinal tumors, and for 85% of appendiceal tumors [**7**]. Females are more affected than males. Carcinoid tumors are found in 0.16 up to 0.7% of all appendectomy specimens [**8,9,27**]. Typically, there is a good long-term prognosis [**26**]. No risk of a secondary neoplasia was proved in long-term studies [**27**]. Romanian reports of appendix carcinoid are very limited [**28**].

There are few cases of appendiceal carcinoids diagnosed during pregnancy, which manifested as appendiceal crises [**10,11**]. A recent case associates an appendiceal carcinoid to an ectopic pregnancy [**12**]. The interaction between the carcinoid tumor of the appendix and the pregnancy has not yet been elucidated [**10**]. The first case diagnosed by transvaginal ultrasound was reported in 2008 [**13**]. Depending on pregnancy age, appendectomy might be performed [**10**]. If an aggressive type of tumor is diagnosed, the second surgical step is hemicolectomy, which may be performed after delivery [**10**]. Sometimes, the tumor is preserved until delivery unless there are signs of local spreading [**5**]. The few reported cases showed no fetal malformations [**14**]. 

One case with incidental appendiceal carcinoid found during a caesarean section was published in the literature. Generally, this surgical procedure as well as all gynecological surgery should include an appendix check-up [**14,15**].

The prognosis of carcinoids depends on several factors including the size and the anatomic location of the tumor, the presence of metastases at diagnosis and the presence of the carcinoid syndrome, the main factor of prognosis in appendiceal carcinoids is the size [**16**]. The cut-off is of 2 cm in diameter [**17**]. Hemicolectomy is indicated in larger tumors, but some doctors avoid it [**18,19**]. Increasing tumor size predicts lymph nodes involvement, whereas age, gender, and depth of tumor invasion do not [**29**]. Regarding the appendix anatomy, 75% of the carcinoids are in the apex, as in our case (with the best prognosis), 20% in the middle portion, and 5% in the base of the appendix [**20,21**]. 

The pathological examination showed insular and alveolar structures, formed by monomorphic round – ovalar cells, with round central nuclei, without prominent nucleoli. Tumoral structures are embedded in a desmoplastic stroma and are localized in the submucosa and muscularis propria, without extension to the serosa. Immunohistochemical investigation confirmed a diffuse positivity of the tumoral cells for chromogranin A and synaptophysin (**Fig 1,2,3**).

In this case, the pathological exam showed the lowest spreading potential, thus no further surgery was necessary [**22,23**]. 

In terms of the surgical procedure, laparoscopic appendectomy (not in the present case) is as safe in the long term, as the conventional type. Pre-operative suspicion of a carcinoid is not a contraindication [**24**]. 

In the present case, after surgery, the neuroendocrine markers were normal, thus the full body octreoscan was not necessary. The follow-up included serum markers and an imagistic scan, performed 3 months after the surgery, then every 6 months, for 1 up to 3 years. Only if necessary, blood tests and imagistic studies can be performed annually thereafter [**25**]. In our case, some authors do not recommend a follow-up. The five years survival rate after surgery is of 71 up to 100% [**16**].

## Conclusion

The carcinoid of the appendix is a rare finding in association with pregnancy. The pathological exam reveals the major aspects regarding the biological behavior and the future necessary therapies. Serial tests of the neuroendocrine markers are the most useful tools in follow-up. Generally, the appendiceal carcinoid is not aggressive. The present case highlights the important role of differential diagnosis of acute abdominal pain in women of childbearing age, and also of rare pregnancy-related appendiceal pathology.

This case is an argue for performing a pregnancy test in all women of childbearing age presenting with acute abdominal pain and also the importance of considering other diagnoses that may mimic or present concurrently with acute appendicitis.

Diagnosis of acute appendicitis is considered more difficult in pregnant than in non-pregnant women. 

The appendicial carcinoid tumor is a lesion that most frequently is discovered incidentally in the removed organ. 

There are only few similar cases were found in the literature reporting appendiceal carcinoid tumor during pregnancy.

Acknowledgments: none 
